# Radiomics-Based Outcome Prediction for Pancreatic Cancer Following Stereotactic Body Radiotherapy

**DOI:** 10.3390/cancers12041051

**Published:** 2020-04-24

**Authors:** Elsa Parr, Qian Du, Chi Zhang, Chi Lin, Ahsan Kamal, Josiah McAlister, Xiaoying Liang, Kyle Bavitz, Gerard Rux, Michael Hollingsworth, Michael Baine, Dandan Zheng

**Affiliations:** 1 Radiation Oncology, University of Nebraska Medical Center, Omaha, NE 68198, USA; elsa.parr@unmc.edu (E.P.); clin@unmc.edu (C.L.); ahsan.kamal@unmc.edu (A.K.); josiah.mcallister@unmc.edu (J.M.); kyle.bavitz@unmc.edu (K.B.); GerardRux@creighton.edu (G.R.); mahollin@unmc.edu (M.H.); 2 Biological Sciences, University of Nebraska Lincoln, Lincoln, NE 68521, USA; qian.du@huskers.unl.edu (Q.D.); zhang.chi@unl.edu (C.Z.); 3 Proton Institute, University of Florida, Jacksonville, FL 32206, USA; XLiang@floridaproton.org

**Keywords:** radiomics, pancreatic cancer, prognosis prediction, SBRT

## Abstract

(1) Background: Radiomics use high-throughput mining of medical imaging data to extract unique information and predict tumor behavior. Currently available clinical prediction models poorly predict treatment outcomes in pancreatic adenocarcinoma. Therefore, we used radiomic features of primary pancreatic tumors to develop outcome prediction models and compared them to traditional clinical models. (2) Methods: We extracted and analyzed radiomic data from pre-radiation contrast-enhanced CTs of 74 pancreatic cancer patients undergoing stereotactic body radiotherapy. A panel of over 800 radiomic features was screened to create overall survival and local-regional recurrence prediction models, which were compared to clinical prediction models and models combining radiomic and clinical information. (3) Results: A 6-feature radiomic signature was identified that achieved better overall survival prediction performance than the clinical model (mean concordance index: 0.66 vs. 0.54 on resampled cross-validation test sets), and the combined model improved the performance slightly further to 0.68. Similarly, a 7-feature radiomic signature better predicted recurrence than the clinical model (mean AUC of 0.78 vs. 0.66). (4) Conclusion: Overall survival and recurrence can be better predicted with models based on radiomic features than with those based on clinical features for pancreatic cancer.

## 1. Introduction

Pancreatic cancer is one of the deadliest cancers, with a one-year survival rate of 20%, and a five-year survival of only 9% [1]. The typical pancreatic adenocarcinoma treatment paradigm for those potentially able to undergo a resection is neoadjuvant chemotherapy followed by surgery [2]. When resection is not initially possible after chemotherapy, radiation may be used with the goal of achieving resectability. While both conventionally fractionated and stereotactic body radiotherapy (SBRT) techniques were used, SBRT gained traction recently due to its favorable side effect profile and convenience of short treatment course [3]. Furthermore, the most compelling trait of SBRT is its suggested superior efficacy, which was shown in the treatment of locally advanced pancreatic adenocarcinoma [4].

A major difficulty in effective pancreatic cancer treatment, however, lies in the heterogeneity of tumors and patients. Unfortunately, the majority of patients do not have any significant reduction in tumor size on imaging after treatment. However, considerable tumor heterogeneity remains among these patients. While some post-treatment tumors remain cancerous with residual disease, others are rid of adenocarcinoma but unchanged in size due to the bulk of the remaining desmoplastic tissue. Current imaging and clinical evaluation methods struggle to distinguish between these tumor responses, making it nearly impossible for clinicians to know at the time of treatment completion if the treatment was successful or if further treatment is necessary. Furthermore, patients also have varying degrees of tolerance to treatment toxicities. Because treatment response and tolerability remain largely unpredictable, there exists a significant need for an evidence-based clinical decision support system to aid physicians in determining the best treatment regimen for each patient.

Radiomics is on the frontier of medical imaging research with clinical use. The radiology/medical imaging analogue of “genomics” and “proteomics”, radiomics uses the vast amount of medical imaging data to extract large numbers of quantitative features to provide valuable tumor information beyond that afforded by the conventional, mostly qualitative image review methods [5,6,7]. While medical images are typically used purely as pictures that provide limited insight into size, shape, and pattern of tumors, radiomic features (such as intensity, shape, texture, or wavelet) could offer additional information associated with cancer phenotype, as well as the tumor microenvironment that is distinct and complementary to other pertinent data sources.

Though in its infancy, radiomics has already proven to be helpful in better understanding the behavior of pancreatic cancer. For example, a 2019 study identified a specific radiomic signature of pancreatic cancer that correlated with overall survival and local control after treatment with stereotactic body radiation therapy [8]. Another study in 2018 analyzed texture features of tumors of the pancreatic head, finding that some features (such as certain filter values and contrast) served as independent prognostic factors in predicting decreased disease free survival [9]. These studies, and others like them, illustrate the ability of radiomic data to provide novel behavioral and prognostic information about individual pancreatic cancers that would not otherwise be discoverable by conventional methods of evaluation.

We are at the advent of uncovering the vast potential of radiomics in providing data-based clinical decision support. In order for radiomics to be a useful clinical decision tool, however, we must first better understand how radiomic data can be used to predict tumor behavior and patient outcomes. Studies such as the aforementioned 2018 Yun et al. study show that radiomic features can be just as useful as clinical features, such as positive lymph node metastasis, in predicting outcomes. However, there is currently a lack of studies that illustrate how radiomic data can provide information and prediction models beyond what our existing prediction models can. In our study, we aimed to use radiomic features of primary pancreatic tumors, in combination with pertinent clinical data, to develop outcome prediction models for pancreatic cancer superior to those using clinical features alone. Specifically, we sought to use radiomic features to predict the overall survival and incidence of local-regional failure of borderline resectable and locally advanced pancreatic cancer patients undergoing stereotactic body radiation therapy (SBRT) following neoadjuvant chemotherapy as a proof-of-concept to determine if such features could outperform currently available clinical information. The incidence of local-regional failure was chosen as an endpoint in addition to overall survival as we consider this currently to be the best surrogate for residual local-regional disease after definitive or neoadjuvant chemoradiotherapy and, as such, an endpoint for which current clinical criteria remains of poor utility.

## 2. Results

**Figure 1:**
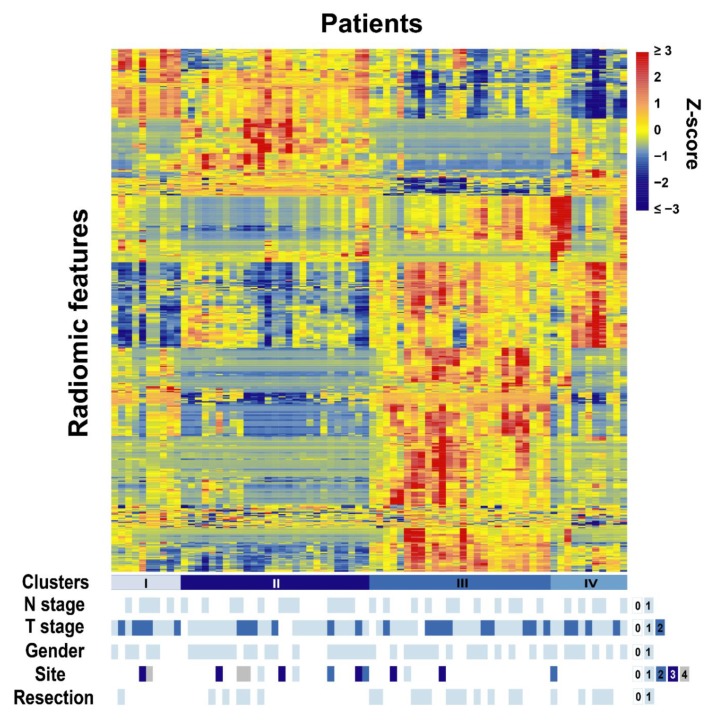
Radiomic heatmap of the studied pancreatic cancer patients. Clinical parameter legend: For N stage, 0 = N0 and 1 = N1; For T stage, 0 = T2, 1 = T3, and 2 = T4; For gender, 0 = female and 1 = male; For Site, 0 = head, 1 = neck, 2 = tail, 3 = body, and 4 = uncinated; For resection, 0 = no and 1 = yes.

Of the 74 patients analyzed in this study, 41 (55.4%) did not have positive lymph nodes, while 33 (44.6%) did. The majority (79.7%) of the patients’ tumors were located in the head of the pancreas, while the remaining 20.3% were in the neck, tail, body, or uncinate. Forty-five (60.8%) of the patients were male, and 29 (39.2%) were female. Of the patients studied, 61 (82.4%) received concurrent chemotherapy with either infusional 5-flourouracil, capecitabine, or nelfinavir, whereas 13 (17.6%) did not receive concurrent chemotherapy. Further information on patient and tumor characteristics can be found in Table 1. With unsupervised clustering, the 74 patients were clustered into four clusters with the radiomic expression patterns and compared against patient clinical parameters (Figure 1). Most of the studied clinical parameters including N stage (*p* = 0.981), T stage (*p* = 0.569), gender (*p* = 0.796), and tumor location (site) (*p* = 0.628) were not significantly associated with patient clusters; only the resection status was associated (*p* = 0.049).

**Figure 2:**
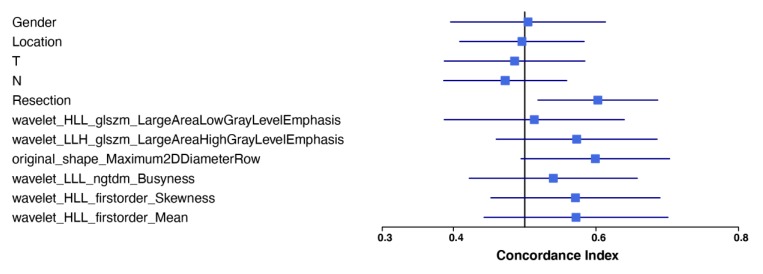
Forest plot of the univariate analysis of the 6 radiomic features chosen for overall survival prediction as well as that of the 5 clinical parameters. The univariate CIs are shown with the 95% confidence interval.

**Figure 3:**
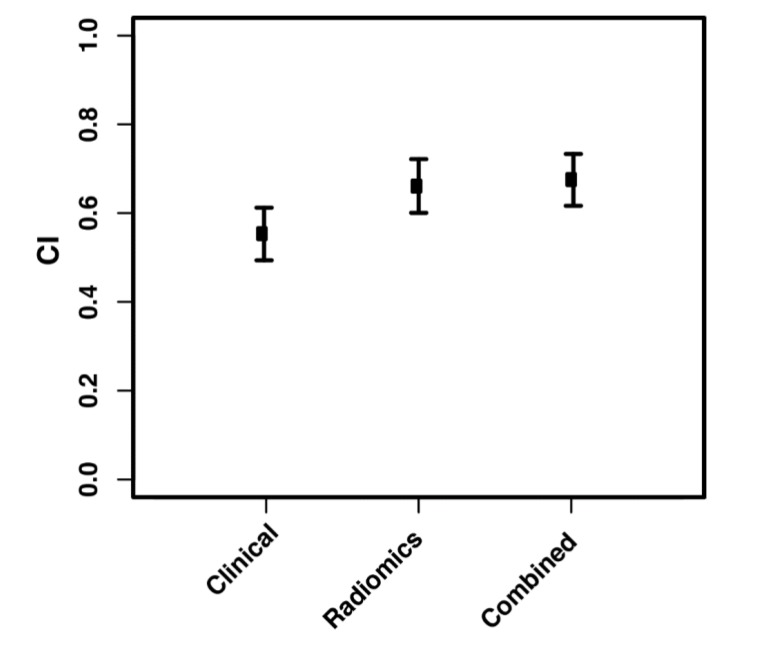
Concordance indices of the survival prediction on the testing datasets achieved using the clinical model, the radiomic model, and the combined model.

**Figure 4:**
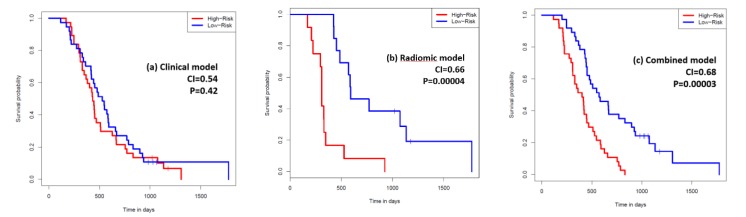
Average Kaplan-Meier survival curves of high- and low-risk patients with pancreatic adenocarcinoima, as predicted by the clinical model (**a**), the radiomic model (**b**), and the combined model (**c**), on the test datasets.

For overall survival, a 6-feature radiomic signature was identified to have the best prediction and was used for the radiomic model. Figure 2 shows the univariate CI of these six radiomic features along with the five clinical features used in our study, all calculated on the whole study population. Comparing the performance of the clinical model, the radiomic model, and the combined model, a mean CI of 0.54, 0.66, and 0.68 was achieved on the 1500 resampled cross-validation test datasets, respectively (Figure 3). The radiomic signature was better at predicting the overall survival than the clinical features. Further combining radiomic and clinical features slightly improved the model performance beyond the radiomic model. Average Kaplan-Meier survival curves comparing the high- and low-risk patients stratified using the median average risk score are shown in Figure 4 to compare the clinical, the radiomic, and the combined model, respectively. The clinical model was unable to predict survival risk with *p* = 0.42 for the log-rank test, while the radiomic and the combined models successfully predicted it with *p* values < 0.0001.

**Figure 5:**
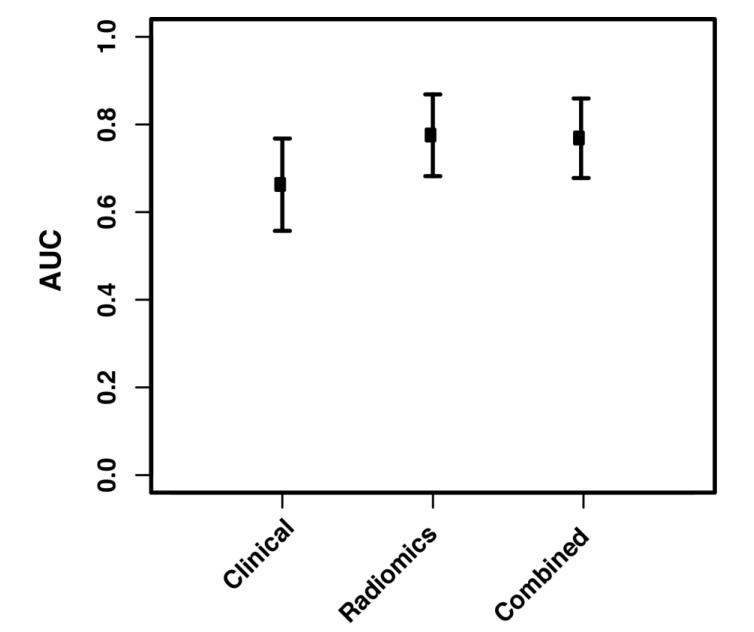
Area under the receiver operating characteristic curve of the recurrence prediction using the clinical model, the radiomic model, and the combined model.

For local-regional recurrence, a 7-feature radiomic signature achieved the best prediction. Table 2 lists these features with their FDR-adjusted *p* values from the univariate ANOVA analysis on the whole study population. Figure 5 compares the performance of the clinical, the radiomic, and the combined model using the area under the receiver operating characteristic curve (AUC) achieved on the 1500 resampled cross-validation test datasets. Mean AUC of 0.66, 0.78, and 0.77 was achieved by the three models, respectively. Again the radiomic signature dominated the recurrence prediction, and the radiomic model outperformed the clinical model. A plot of the area under the precision-recall curve (AUPRC) results is also presented in the Supplementary Figure S1 to compare the three models, with mean AUPRCs of 0.51, 0.67 and 0.69, respectively.

## 3. Discussion

In this study, we extracted and analyzed radiomic data from the CT simulation scans of 74 borderline resectable and locally advanced pancreatic cancer patients to develop better outcome prediction models. All images were acquired on a single scanner with the same acquisition protocol including acquisition technical parameters and the contrast injection protocol. This aspect of imaging data homogeneity helped reduce the radiomics data uncertainty.

For pancreatic cancer, contrast-enhanced CT is the most important diagnostic and monitoring imaging modality, routinely used in the entire patient management course for workup and follow-up. These available images provide an environment ideal for machine learning and data-based science. Radiomic data harvested from the imaging promises to provide valuable patient- and tumor-specific heterogeneity information, which can better inform longitudinal changes in tumor features and behavior. Radiomics thus holds the potential to be a robust source of information used for evidence-based clinical decision making.

We used radiomics to identify signatures that are superior to clinical features in predicting overall survival and local-regional recurrence.

A recent retrospective case-control study also used radiomic data from CT scans to assess pancreatic cancer [10]. The study identified patients with pancreatic ductal adenocarcinoma and compared the radiomic features of their pancreases to those of healthy renal donors. The researchers extracted 478 radiomic features, forty of which they used to differentiate the healthy and cancerous pancreases. Using radiomic features alone, the researchers were able to correctly identify all 60 cases of adenocarcinoma, and only mislabeled one healthy pancreas as cancerous. This study, like ours, shows the ability of radiomics alone to provide key information about pancreatic tissue. However, while the analysis demonstrated the usefulness of radiomics in the diagnosis of pancreatic cancer, it did not delve into its ability to predict prognosis.

A later study published in 2019 also used radiomics to analyze pancreatic adenocarcinoma tumors [8]. This study focused on prognosis prediction. Researchers identified a specific combined clinical-radiomic signature that correlated with overall survival and local control of pancreatic carcinoma after treatment with SBRT (*p* = 0.05 and 0.004, respectively). Like our study, this study illustrated how specific radiomic features may relate to prognosis. However, this study did not address whether the model performance was driven by the clinical or the radiomic factors included in the models. In addition, only a small panel of radiomic features, 41 texture features, were investigated in this study, and the study applied a random data split into training and validation datasets without performing cross-validation or resampling to assess the possible overfitting uncertainty of the results.

A 2018 study analyzed the ability of both radiomic and clinical data to predict overall survival [9]. They found that the presence of lymph node metastasis was an independent factor associated with disease free survival (hazard ratio = 1.957 to 2.181, depending on the filter applied). They also found that various radiomic features, such as contrast, independently correlated with disease free survival (HR = 0.4665). While the study demonstrated the capability of correlating both clinical features and individual radiomic features with survival, it did not compare the two. This is a notable difference from our study, which found that the combined radiomic and clinical data could better predict survival than the clinical data alone, and the superior performance was primarily due to the contribution of the radiomic signature.

While radiomics has already had a variety of applications in many different cancers, and been integrated into routine clinical practice as computer-aided diagnostic tools for some, its usefulness in pancreatic cancer remains less explored but desperately needed [11]. As an extremely lethal cancer with the highest mortality rate of all major cancers in the US [12], pancreatic cancer is a critical global health care problem. Any potential benefit radiomics can provide beyond the current clinical systems will be meaningful, no matter in the realms of early detection, diagnosis, or treatment decision making. For the latter, TNM staging is currently at the core. However, it relies heavily on gross anatomy and pathology, making it a less detailed and more invasive method of evaluation. In contrast, radiomics is noninvasive and can reveal details of tumor heterogeneity, thus providing more easily accessible and detailed information that can be longitudinally tracked for superior prediction of treatment outcome. Our study shows a proof-of-concept in which radiomics exceeds the abilities of its more traditional counterparts in the understanding and prediction of tumor behavior to better inform and treat patients with this deadly disease.

Another application of radiomics not explored by our study is the use of delta-radiomics to assess treatment response [13]. By comparing the differences in patients’ longitudinal radiomic data as they progress through treatment, researchers were able to assess treatment response earlier and more reliably than current methods of assessment, such as trending CA-19-9, in pancreatic cancer [14]. A recent study performed by Nardone et al. further suggests that the use of radiomics in tracking treatment response is better accomplished by the analysis of delta-radiomics than of a single radiomic dataset [15]. Thus, the use of delta-radiomics creates a unique potential for physicians to adapt treatment plans to patients’ treatment response earlier and more accurately than currently possible. Because CT imaging is routinely used in pancreatic cancer for daily setup during SBRT treatment courses and for longitudinal follow-up, it will be an interesting future investigation to further extend our findings to evaluate the additional benefit of delta-radiomics.

The findings of our study are not without limitations. The study was a single institution study with a relatively small patient number. Due to this limited patient population, we were unable to correlate our results with other patient factors, such as distant failure, time to recurrence, and response to various other treatment regimens. The patients were not all treated with a homogenous radiation dose, which may not have been fully addressed in our study. Also, though the segmentation uncertainty likely plays an important role in radiomic modeling, our study was not designed to investigate or mitigate such uncertainties, such as using a novel double manual contouring method described in Nardone et al.’s work [16]. On the other hand, efforts were made in this study to reduce the uncertainty by post-editing the clinical tumor contours (GTVs) and excluding the uncertain areas as described in the methods.

The results of our study show promise for the future of radiomics and its ability to provide new, superior information for evidence-based clinical decision making in pancreatic cancer. While these results are encouraging, it should be noted that they provide only a preliminary investigation and require further external validation. In this work, we attempted various ways to maximize the use of the limited data, such as performing extensive resampling in feature selection and cross-validation steps, to mitigate potential overfitting and enhance the robustness of our results. On the other hand, part of our study design still allowed potential for information leak due to the lack of an independent testing dataset. Specifically, although the holdout set was unseen during the model fitting process, the holdout data was included in the feature selection steps of the pipeline. Also the data normalization was performed on the whole dataset, permitting another potential source of information leak. Limitations withstanding, our proof-of-concept study illustrated the promise of improving pancreatic cancer prognosis prediction via the synergy of radiomics and clinical models. The results should be validated via the expansion of the dataset, preferably via a multi-institutional study. Further analysis should include studies on how radiomic features can predict control patterns and patient resectability, as well as how radiomic patterns can change longitudinally with various treatment regimens.

## 4. Materials and Methods

### 4.1. Patient Selection and Treatment Information

Patient demographic, treatment, and outcome data were collected from electronic medical records of patients with pancreatic adenocarcinoma between 2007 and 2016. A total of 74 patients were analyzed in the study. Data were collected for all patients who underwent SBRT at a single academic medical center. Information gathered included patient’s date of birth, date of diagnosis, gender, tumor location, TNM stage at diagnosis, dates of radiation delivery, and any systemic therapy delivered concurrently with the respective radiation treatments. The contrast-enhanced CT images used for SBRT treatment simulation were also collected for radiomic data extraction. All data collection was approved by the IRB of our institution (Protocols: 728-16-EP and 127-18-EP).

All patients were treated with neoadjuvant chemotherapy and were without evidence of disease progression prior to SBRT. For SBRT, all patients received 25–40 Gy in five fractions with fiducial markers to localize and track the tumor. Radiation was primarily delivered to the intact pancreas tumors alone with a singular exception consisting of a patient who had a Whipple procedure with a focally identifiable positive margin which was subsequently treated with SBRT. All patients also underwent further chemotherapy following SBRT completion. Chemotherapy regimens were at the discretion of the treating medical oncologist. As most patients were treated by community medical oncologists and were referred to our institution for radiation oncology and surgical evaluation only, details regarding chemotherapy timing and use of specific chemotherapy regimens were often not available to our institution.

### 4.2. Assessment of Patient Outcomes

Following completion of SBRT, patient electronic medical records were assessed including both clinical notes and results from follow-up imaging until the date patients were either lost to follow-up or succumbed to their disease.

Overall survival was calculated from time of the SBRT simulation CT scan to date of death. Date of death was obtained through either documented date of death in the patient’s respective electronic medical record or through publicly available information from obituaries. Patients who were lost to follow-up were censored at the time of last follow-up.

To assess disease local-regional recurrence, we used radiological findings from follow-up computed tomography (CT) scans of the abdomen and pelvis and from magnetic resonance imaging scans of the abdomen. The scans used were performed after the delivery of radiation therapy. We defined recurrence as persistence on two consecutive scans, per the Response Evaluation Criteria in Solid Tumors version 1.1 [17]. The date of failure was then changed to the date of the first scan that demonstrated evidence of failure.

### 4.3. Tumor Segmentation and Feature Extraction

For the SBRT simulation CT, all patients underwent a contrast-enhanced free-breathing CT scan of the abdomen. The scans were acquired using the same protocol for all patients in 2 mm axial slice thickness with the same Sensation Open CT simulator (Siemens, Erlangen, Germany). To improve segmentation consistency, using the gross tumor volume of SBRT as a starting point, each tumor was manually re-segmented using a consistent window/level setting from the contrast-enhanced CT for this study, excluding any area of uncertainty. From each segmented tumor, 841 radiomic features were extracted using the radiomics module on 3D Slicer 4.9 and visualized using an interactive visualization platform [18,19]. A resampled 2 × 2 × 2 mm^3^ voxel size and a bin width of 25 were used for feature extraction. The features are defined in compliance with feature definitions as described by the Imaging Biomarker Standardization Initiative (IBSI) and can be divided into original features (105 features) and wavelet features (736 features) [20]. The original features can be subdivided into 7 classes, including 13 Shape features, 18 First Order statistical features, 23 Gray Level Co-occurrence Matrix (GLCM) features, 14 Gray Level Dependence Matrix (GLDM) features, 16 Gray Level Run Length Matrix (GLRLM) features, 16 Gray Level Size Zone Matrix (GLSZM) features, and 5 Neighboring Gray Tone Difference Matrix (NGTDM) features. The wavelet features included all except Shape features calculated on the filtered images with all 8 combinations of applying either a High or a Low pass filter in each of the three dimensions. Feature data were normalized over the whole dataset before further data analysis.

### 4.4. Data Analysis

**Figure 6:**
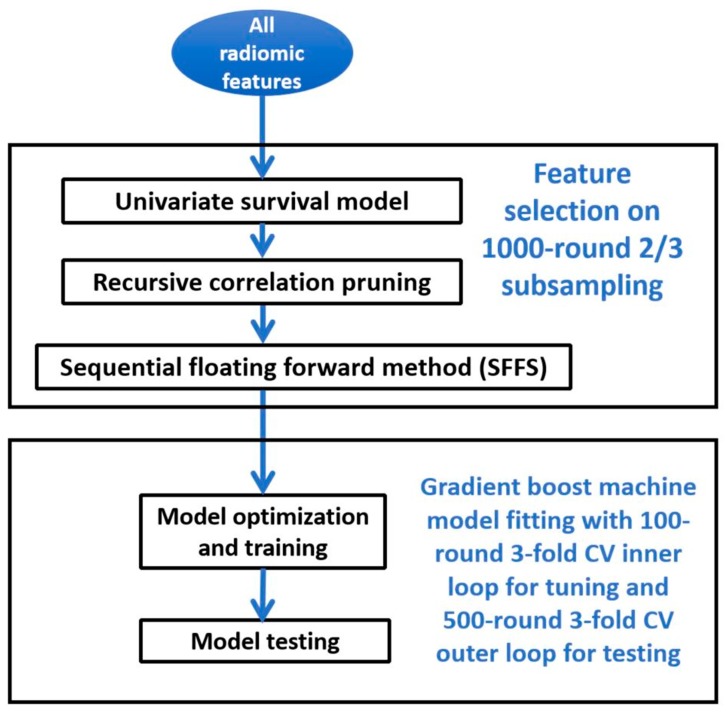
Radiomic analysis workflow.

A heatmap was first generated using unsupervised clustering to investigate the radiomic feature patterns as well as their relationship with clinical parameters including N stage, T stage, gender, tumor location (site), and the resection status. The clustering was conducted with average-linkage based on the Euclidean distance for both features and patients. The associations between patient clusters and the clinical parameters were tested using a χ^2^ test of independence for each parameter. Subsequently, radiomic features were used to predict the two clinical endpoints, overall survival (calculated from the date of the SBRT simulation CT) and local-regional recurrence. For both endpoints, the basic workflow of the radiomic analysis consists of feature selection steps and modeling/testing steps depicted in Figure 6. The overall survival prediction was evaluated using the Harrell concordance index (CI), and the recurrence prediction was evaluated using the AUC and AUPRC [21,22]. For radiomic feature selection, a 3-step process was performed including a univariate analysis to select the relevant features, a recursive correlation pruning step to remove redundant features, and a sequential floating forward method (SFFS) to determine the best feature combination. All data analysis, described with more details in the following sub-sections, was performed using R (version 3.3.2) [23].

#### 4.4.1. Overall Survival Prediction

For overall survival, the univariate analysis applied the Cox proportional hazard model. The false discovery rate (FDR)-adjusted *p*-value from the univariate analysis was used for the first step of feature selection followed by recursive correlation pruning and SFFS. A 1000 times data resampling was applied in the feature selection process, each time using randomly resampled 2/3 of the data. The prediction model used was an ensemble model of Cox proportional hazard models trained by a gradient boosting machine algorithm in order to optimize the prediction performance measured by CI. The performance of the model was evaluated by nested resampling with an outer loop of 500 times 3-fold cross-validations to evaluate the effect of random sampling on the model performance and an inner loop of 100 times 3-fold cross-validations to optimize the model hyperparameters. The 1500 (3 × 500) CI values calculated from the outer loop cross-validation test datasets were used to describe the performance of the prediction model. A workflow schematic is presented in Supplementary Figure S2 for the nested resampling.

Three models were created for the survival prediction. The clinical model used clinical features only (gender, T stage, N stage, site, and resection). The radiomic model used radiomic features only, following the feature selection and modeling process described above. The combined model combined all five clinical features with the selected radiomic features. The performance of the three models were compared using the mean value and the dispersion of the 1500 CI values.

#### 4.4.2. Local-Regional Recurrence Prediction

To select features for recurrence prediction, 2/3 of the total samples were used without replacement. Except using the ANOVA analysis instead of the Cox hazardous model in the univariate step, the rest of the feature selection steps were the same as in the overall survival prediction analysis. A 1000 times resampling was applied in the SFFS step to mitigate overfitting.

The recurrence prediction model also used the gradient boosting machine model and applied a nested resampling similar to described above for the overall survival prediction. For evaluation, the 1500 AUC values from the outer cross-validation loops were used. Similar to described above, three models were compared with the AUC results: the clinical model, the radiomic model, and the combined model.

## 5. Conclusions

Radiomic data can be used to better understand tumor behavior and create improved prediction models. In this study, we compared radiomics-based prediction models with traditional clinical prediction models to assess their ability in predicting pancreatic cancer outcomes. We identified specific radiomic signatures that were superior to clinical features in prognosis prediction. These results encourage further research on how radiomic data can be used to enhance our understanding of other aspects of tumor behavior and better inform treatment.

## Figures and Tables

**Table 1:** Patient and treatment characteristics.

Characteristic	Number of Patients (Percentage)
Gender	
Male	45 (60.8%)
Female	29 (39.2%)
Median age (range) Tumor site in pancreas	62 (34–86)
Head	59 (79.7%)
Neck	3 (4.1%)
Tail	3 (4.1%)
Body	6 (8.1%)
Uncinate	3 (4.1%)
N stage	
0	41 (55.4%)
1	33 (44.6%)
T stage	
2	5 (6.8%)
3	44 (59.5%)
4	25 (33.8%)
Use of SBRT ^a^	
Definitive	51 (68.9%)
Neoadjuvant	23 (31.1%)
Concurrent chemotherapy	
None	13 (17.6%)
Infusional 5-FU ^b^	4 (5.4%)
Capecitabine	11 (14.9%)
Nelfinavir	46 (62.2%)
Survival	
Alive	5 (6.8%)
Deceased	69 (93.2%)
Median overall survival (months (95% CI ^c^))	
From diagnosis	15 (14–17)
From SBRT	11 (10–14)
Days since diagnosis (alive patients)	116–1776
Median days to death (recorded deaths)	452.5

^a^ SBRT—stereotactic body radiation therapy; ^b^ 5-FU—5-fluorouracil; ^c^ CI—confidence interval.

**Table 2:** Radiomic features selected for the recurrence prediction model and their corresponding univariate FDR-adjusted *p* values.

Feature	FDR-Adjusted *p* Value
wavelet_HLH_glszm_SmallAreaEmphasis	0.004
wavelet_HLL_firstorder_Kurtosis	0.050
wavelet_HHH_gldm_DependenceNonUniformityNormalized	0.098
wavelet_HHL_gldm_SmallDependenceHighGrayLevelEmphasis	0.029
wavelet_HHH_firstorder_Skewness	0.166
wavelet_LLL_glcm_Correlation	0.152
wavelet_HHL_glrlm_ShortRunHighGrayLevelEmphasis	0.028

## Supplementary Materials

The following are available online at https://www.mdpi.com/2072-6694/12/4/1051/s1, Figure S1: Area under the precision-recall curve (AUPRC) of clinical, radiomic, and combined clinical + radiomic features; Figure S2: Data resampling and cross-validation in the developed radiomic model workflow.
