# Supplementary material for: Radiomics-Based Outcome Prediction for Pancreatic Cancer Following Stereotactic Body Radiotherapy
Source: Cancers (Basel). 2020 Apr 24;12(4):1051. doi: 10.3390/cancers12041051 (PMC7226523; doi:10.3390/cancers12041051)
Supplement: Supplementary file 1 [file cancers-12-01051-s001.pdf]

cancers

MDPI

Supplementary Materials:

Radiomics-Based Outcome Prediction for Pancreatic Cancer Following Stereotactic Body Radiotherapy

Elsa Parr [1,†], Qian Du [2,†], Chi Zhang [2], Chi Lin [1], Ahsan Kamal [1], Josiah McAlister [1], Xiaoying Liang [3], Kyle Bavitz [1], Gerard Rux [1], Michael Hollingsworth [1], Michael Baine [1,*] and Dandan Zheng [1,*]

[1] Radiation Oncology, University of Nebraska Medical Center, Omaha, NE 68198, USA; elsa.parr@unmc.edu (E.P.); clin@unmc.edu (C.L.); ahsan.kamal@unmc.edu (A.K.); josiah.mcallister@unmc.edu (J.D.); kyle.bavitz@unmc.edu (K.B.); GerardRux@creighton.edu (G.R.); mahollin@unmc.edu (M.H.)

[2] Biological Sciences, University of Nebraska Lincoln, Lincoln, NE 68521, USA; qian.du@huskers.unl.edu (Q.D.); zhang.chi@unl.edu (C.Z.);

[3] Proton Institute, University of Florida, Jacksonville, FL 32206, USA; XLiang@floridaproton.org

† Co-first-author, contributed equally

* Correspondence: mbaine@unmc.edu (M.B.); dandan.zheng@unmc.edu (D.Z)

Received: 24 March 2020; Accepted: 22 April 2020; Published: 24 April 2020

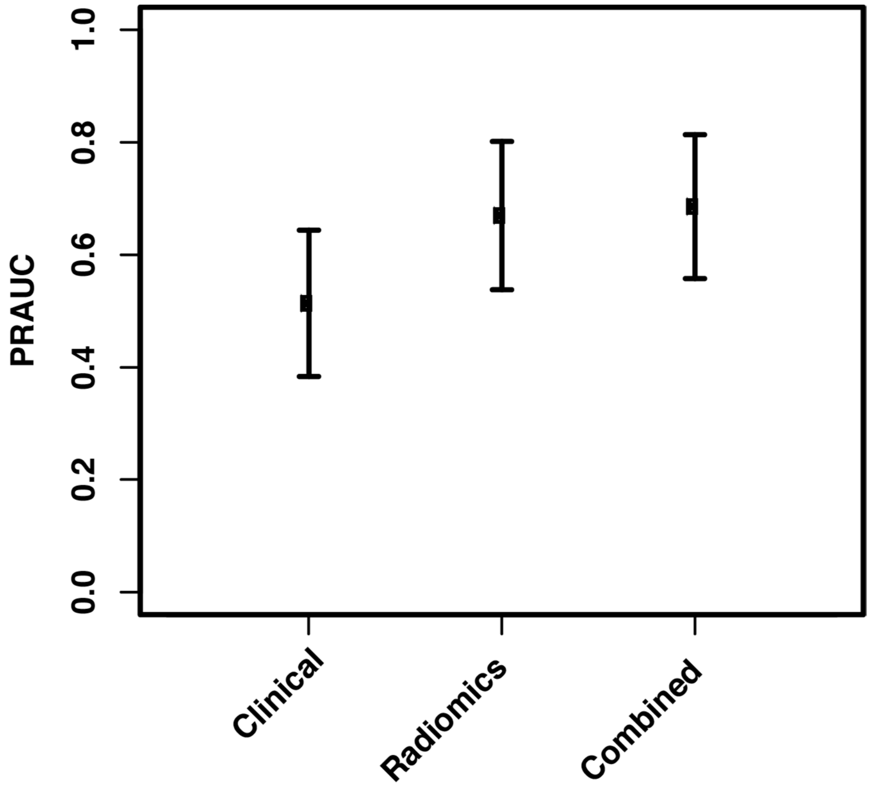

Figure S1. Area under the precision-recall curve (AUPRC) of clinical, radiomic, and combined clinical + radiomic features.

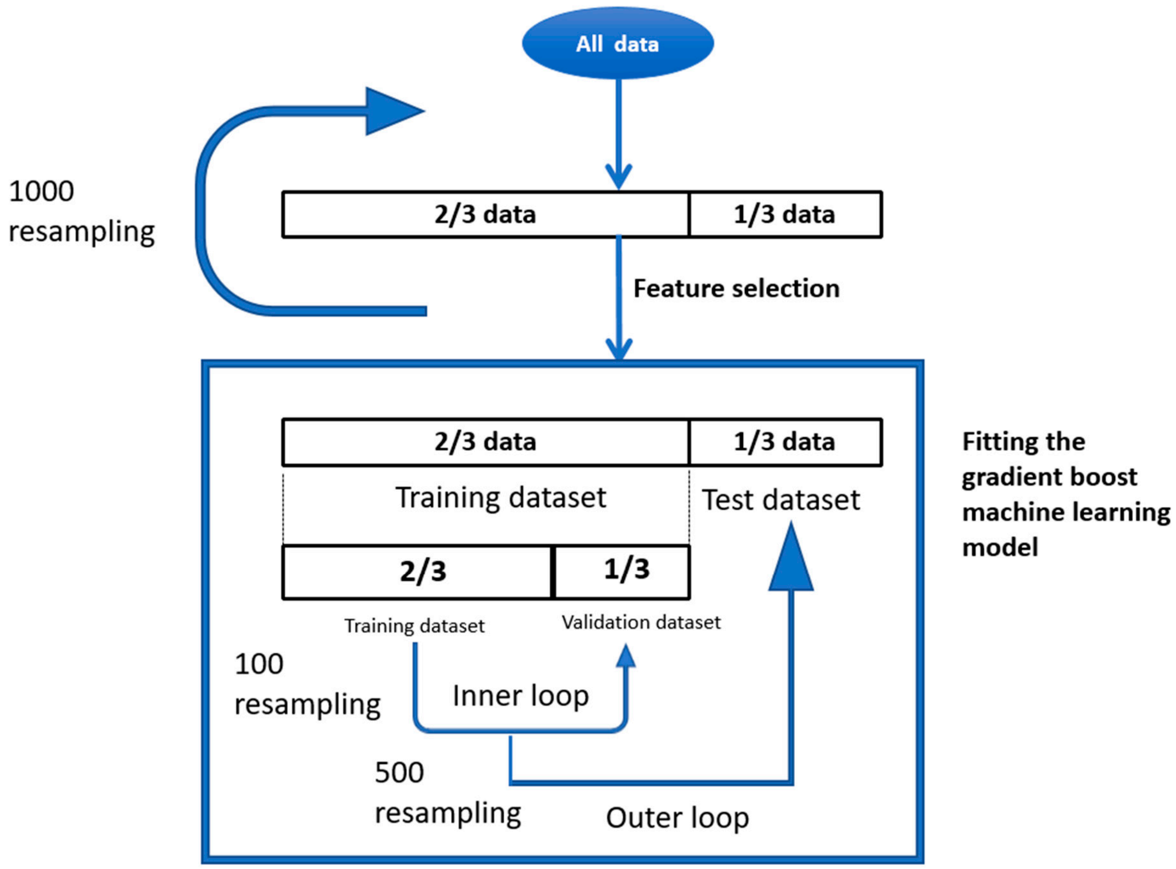

Figure S2. Data resampling and cross-validation in the developed radiomic model workflow.
